# Organic‐Inorganic Hybrid Solid Composite Electrolytes for High Energy Density Lithium Batteries: Combining Manufacturability, Conductivity, and Stability

**DOI:** 10.1002/advs.202406774

**Published:** 2024-11-14

**Authors:** Dries De Sloovere, Jonas Mercken, Jan D'Haen, Elien Derveaux, Peter Adriaensens, Philippe M. Vereecken, Marlies K. Van Bael, An Hardy

**Affiliations:** ^1^ Institute for Materials Research (imo‐Imomec) UHasselt and Imec Agoralaan, Building D Diepenbeek 3590 Belgium; ^2^ EnergyVille Thor Park 8320 Genk 3600 Belgium; ^3^ Imec Energy Department Kapeldreef 75 Leuven 3001 Belgium; ^4^ KULeuven M2S, cMACS Celestijnenlaan 200F Leuven 3001 Belgium

**Keywords:** ionogel, lithium‐ion battery, organosilane, potential

## Abstract

The deployment of solid and quasi‐solid electrolytes in lithium metal batteries is envisioned to push their energy densities to even higher levels, in addition to providing enhanced safety. This article discusses a set of hybrid solid composite electrolytes which combine functional properties with electrode compatibility and manufacturability. Their anodic stability >5 V versus Li^+^/Li and compatibility with lithium metal stem from the incorporated ionic liquid electrolyte, whereas the organic‐inorganic hybrid host structure boosts their conductivity up to 2.7 mS cm^−1^ at room temperature. The absence of strong acids enables compatibility with porous NMC811 electrodes. Liquid precursor solutions can be readily impregnated into porous electrodes, facilitating cell assembly. Electrolytes containing TFSI^−^ as the only anion have a superior compatibility toward high‐voltage positive electrode materials, whereas electrolytes containing both FSI^−^ and TFSI^−^ have a better compatibility toward lithium metal. Using the former as catholyte and the latter as anolyte, NMC811/Li coin cells retain up to 100% of their initial capacity after 100 cycles (0.2 C, 3.0–4.4 V vs Li^+^/Li). Because of their unprecedented combination of functional properties, electrode compatibility, and manufacturability, these hybrid solid composite electrolytes are potential candidates for the further development of lithium metal battery technology.

## Graphical abstract

This works explores hybrid solid composite electrolytes that offer functional properties, electrode compatibility, and manufacturability. Liquid and acid‐free precursor solutions can be easily impregnated into electrodes, enabling electrode/electrolyte compatibility and simplifying cell assembly. NMC811/Li coin cells maintain up to 100% capacity after 100 cycles, making these electrolytes promising candidates for advancing lithium metal battery technology.

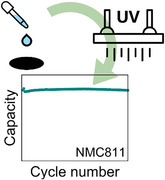

## 1 Introduction

The use of solid or quasi‐solid electrolytes in lithium batteries instead of their liquid counterparts allows to maximize the amount of active material in each cell, increasing energy density. Also, such electrolytes may allow the efficient use of lithium metal, which has the highest theoretical capacity (3860 mAh g^−1^) and lowest redox potential (−3.04 V vs the standard hydrogen electrode) of all potential negative electrode materials.^[^ ^1^, ^2^ ^]^ Further, the absence of flammable carbonate solvents implies that the use of these electrolytes can mitigate safety issues.^[^ ^3^ ^]^

Whether they are liquid or solid, battery electrolytes should have a high ionic conductivity and a wide electrochemical stability window to provide (electro)chemical compatibility with both electrodes. In practice, most electrolytes will be reduced when contacted with lithium metal and oxidized in contact with a high‐voltage positive electrode material such as LiNi_0.8_Mn_0.1_Co_0.1_O_2_ (NMC811). Ideally, this should lead to the formation of passivating interfaces, which mitigate further electrolyte breakdown because of their electronic isolation, but which still allow the transport of lithium ions to enable stable electrochemical operation.^[^ ^4^ ^]^ As such, the formation of a robust solid‐electrolyte interphase (SEI) is crucial for the stable operation of lithium metal negative electrodes.^[^ ^5^ ^]^ Conventional liquid electrolytes are based on the dissolution of LiPF_6_ in organic carbonate solvents. Whereas this system provides excellent compatibility with graphite‐based negative electrodes, the unavoidable presence of water traces leads to the formation of HF, which in turn causes transition metal dissolution from the positive electrode and destroys the SEI formed on the negative electrode.^[^ ^6^ ^]^ Ionic liquid electrolytes (ILEs) can provide a possible alternative for conventional liquid electrolytes, as they avoid the formation of HF. In addition, ILEs have a high ionic conductivity and a broad electrochemical stability window, are thermally stable, and are considered nonflammable.^[^ ^7^, ^8^ ^]^ Different IL anions can be mixed to achieve the desired functional properties. For instance, Kerner et al. showed that mixing bis(trifluoromethanesulfonyl)imide (TFSI^−^)‐ and bis(fluorosulfonyl)imide (FSI^−^)‐based ILs allows to combine the former's higher anodic stability with the latter's improved compatibility with lithium metal.^[^ ^7^ ^]^ The decomposition of LiFSI on lithium metal leads to the formation of a stable SEI rich in LiF, positively influencing the reversibility of the lithium plating/stripping process.^[^ ^9^ ^]^

ILEs can be incorporated in a solid matrix, forming a solid composite electrolyte (SCE), which can be more specifically referred to as ionogel.^[^ ^3^ ^]^ Here, the confined ILE becomes solid or solid‐like due to its interaction with the host matrix. As such, the ILE is endowed with the advantages inherent to solid electrolytes. To be commercially viable, SCEs should be designed for manufacturability. This can be achieved by liquid processing, where a precursor solution is impregnated in a porous electrode and is then instantaneously transformed into the SCE, resulting in an intimate electrode/electrolyte contact.^[^ ^10^ ^]^ Polymer‐based SCEs typically have a good manufacturability, but generally have a low ionic conductivity.^[^ ^11^, ^12^, ^13^, ^14^, ^15^, ^16^, ^17^ ^]^ Higher conductivity values can be achieved for silica‐based SCEs. These are conventionally synthesized in a non‐hydrolytic route which relies on the addition of acids (formic acid,^[^ ^18^ ^]^ HCl,^[^ ^19^ ^]^ HPF_6_,^[^ ^20^ ^]^ or acetic acid^[^ ^21^ ^]^) to form the matrix in a short timeframe. Unfortunately, the use of acids leads to electrode incompatibility, as the acids damage the active material (for instance by inducing transition metal dissolution).^[^ ^3^, ^22^ ^]^ Alternatively, silica‐based SCEs can also be formed in a hydrolytic synthesis route which in principle does not require the addition of acids, enabling electrode compatibility.^[^ ^23^ ^]^ However, this is a spontaneous process, meaning that solidification will occur at a certain time after mixing the reagents. Moreover, the liquid precursor takes several days to completely solidify, resulting in poor manufacturability.

Here, we describe a novel type of hybrid SCE, which combines functional properties with electrode compatibility and manufacturability. Liquid precursor solutions with indefinite stability were developed by combining two alkoxysilanes in a solution which also contains an ILE, water, and a solvent. The vinylidene moieties on one of the alkoxysilanes can be crosslinked by rapid photo‐initiated radical polymerization, effectively creating an organic‐inorganic hybrid solid matrix which encapsulates the ILE. These precursor solutions were impregnated into porous electrodes and instantaneously solidified. In this manner, the SCEs were used as solid electrolytes in NMC811/Li cells. By varying the ILE composition of the SCEs, their compatibility toward either electrode could be emphasized. Combining functional properties, electrode compatibility, and manufacturability, this versatile set of hybrid SCEs is a candidate for the further development of lithium metal batteries.

## 2 Results

**Figure 1:**
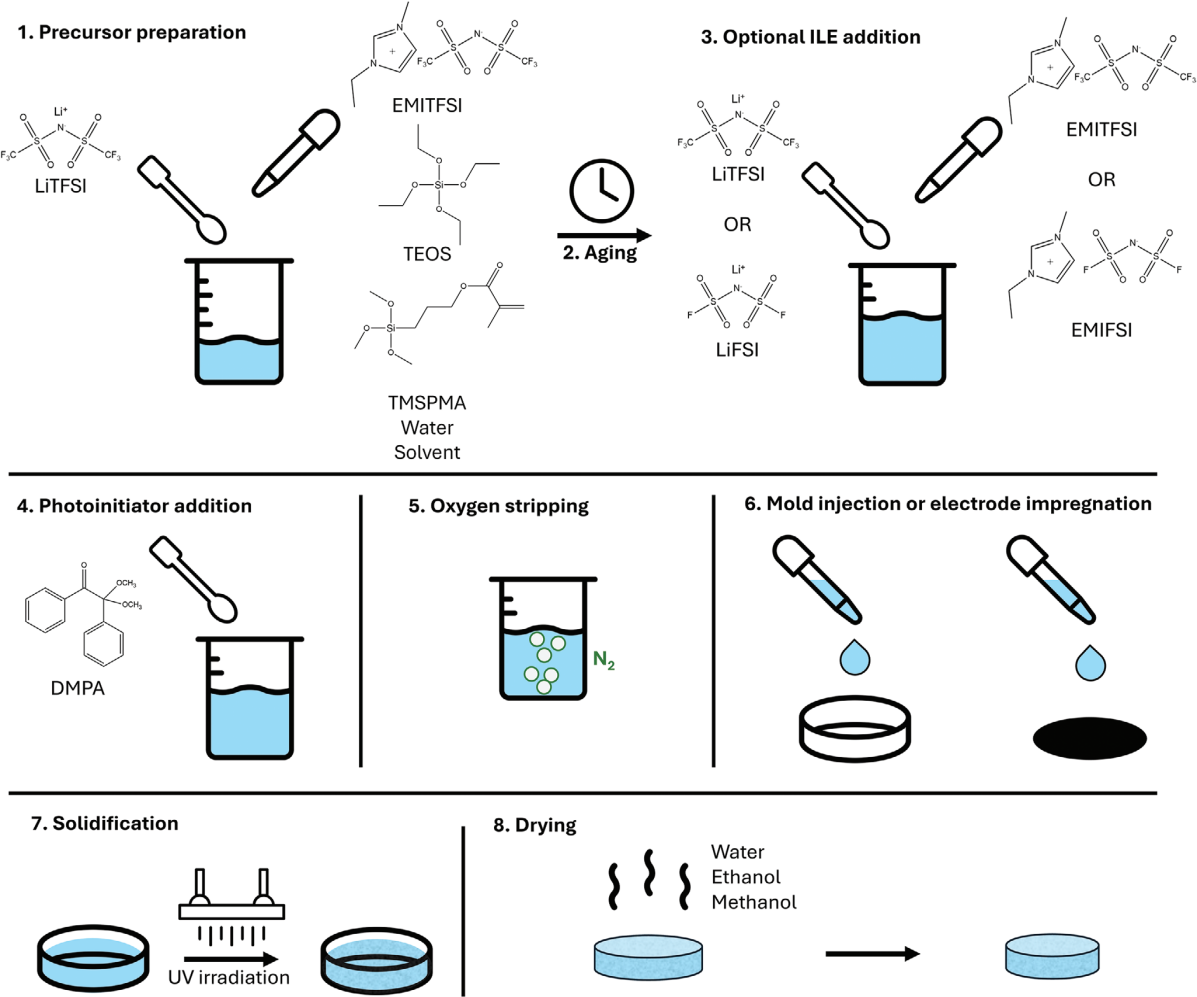
Schematic representation of the SCE synthesis procedure.

The necessity to combine high ionic conductivity with good manufacturability implies that a silica‐based SCE must be formed within a short timeframe after application of an external trigger and without the addition of acids that may damage the electrodes. Therefore, we set out to modify the conventional hydrolytic synthesis process of silica‐based SCEs to such an extent that spontaneous solidification is prevented and is only possible when the solution is subjected to an external trigger. This process, outlined in **Figure** **1**, is discussed in detail in the following section.

### 2.1 Synthesis Procedure

#### 2.1.1 Precursor Preparation and Aging

Precursor solutions were prepared by mixing an ILE with the reagents for the solid matrix. A 1:3 molar ratio LiTFSI:EMITFSI (1‐ethyl‐3‐methylimidazolium bis(trifluoromethylsulfonyl)imide) solution was chosen as ILE, as the TFSI^−^ anion has a superior chemical stability against hydrolysis.^[^ ^24^ ^]^ The reagents for the solid matrix consisted of 3‐(trimethoxysilyl)propyl methacrylate (TMSPMA) and tetraethyl orthosilicate (TEOS) as matrix precursors, excess water to allow the hydrolysis of the alkoxy groups, and an alcoholic solvent. TEOS contains four alkoxy groups per silicon atom and is therefore able to form four oxo‐bridges with other matrix precursor molecules (TEOS or TMSPMA). TMSPMA contains three alkoxy groups per silicon atom, and can therefore form three oxo‐bridges with other matrix precursor molecules.

The precursor solutions were aged at room temperature, in a closed environment, and without stirring. As mentioned in a previous report, solidification could be observed within a matter of days in precursor solutions containing solely TEOS as silica source.^[^ ^23^ ^]^ Hydrolysis and condensation of TEOS, believed to be catalyzed by the Li^+^ ions and influenced by the ILE as template, resulted in a solidification process of 5–9 days, depending on the exact composition. In our case, a range of precursor solutions was prepared by keeping the concentration of matrix precursor molecules (i.e., [Si], equal to [TEOS] + [TMSPMA]) constant, while varying their ratio. Increasing the TMSPMA content of the precursor solution progressively increased the time after which spontaneous solidification was observed. At [TMSPMA]:[Si] ratios of 50% or higher, the spontaneous solidification process was completely avoided, i.e., the precursor solution remained liquid for an indefinite amount of time. Based on these observations, two precursor solutions were defined (**Table** **1**), containing as matrix source either solely TMSPMA (solution A), or an equimolar ratio of TEOS and TMSPMA (solution B). The [EMITFSI]:[Si] ratio of both solutions was 1.5:1. The detailed compositions of the precursor solutions are given in Table 1.

**Table 1:** The contents of precursor solutions A and B.

	Solution A	Solution B
LiTFSI [mmol]	5.67	5.67
EMITFSI [mmol]	17.0	17.0
TEOS [mmol]	0	5.65
TMSPMA [mmol]	11.3	5.65
Water [mL]	2.5	2.5
Solvent [mL]	5	5

**Figure 2:**
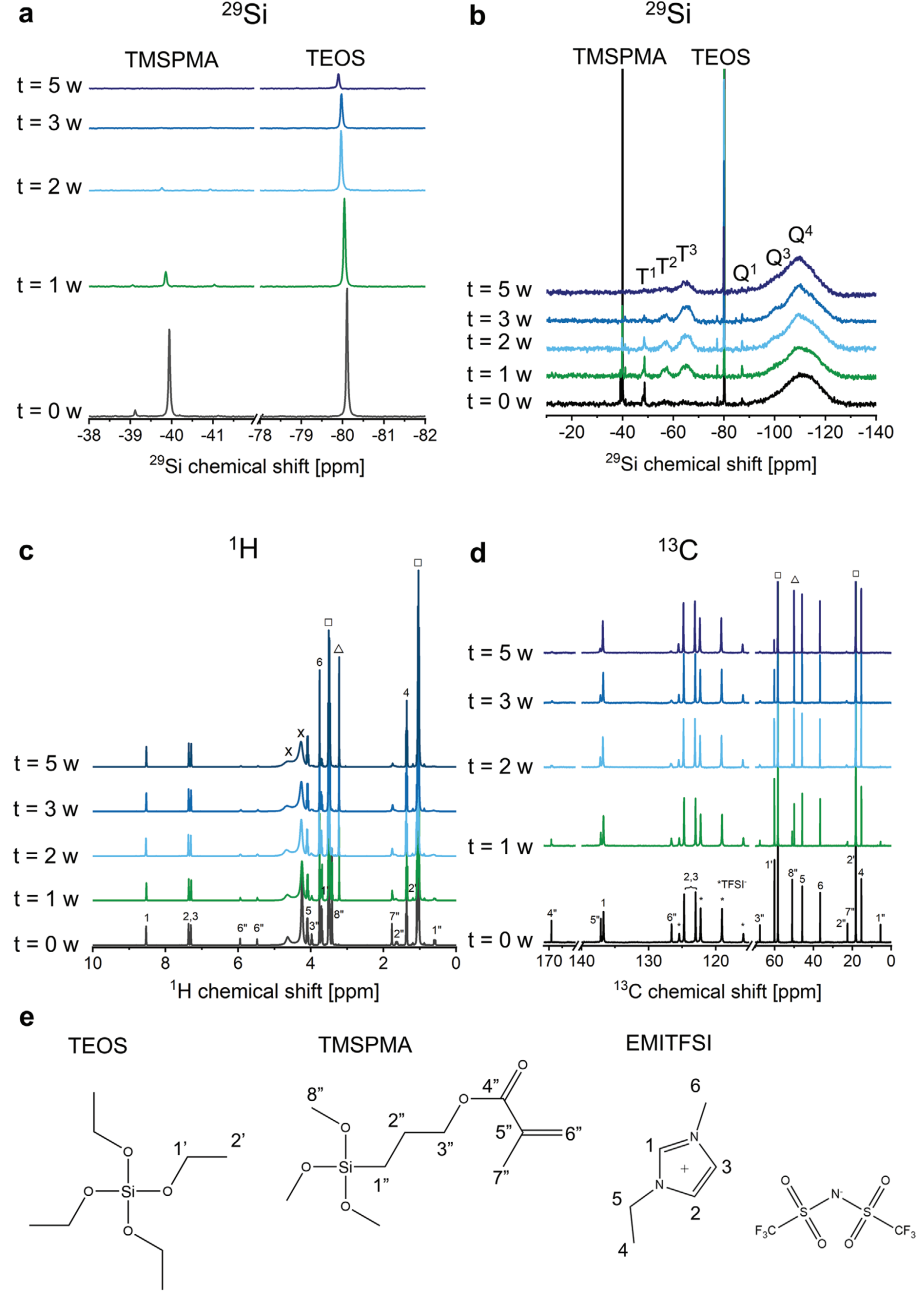
TMSPMA and TEOS undergo hydrolysis and condensation reactions after mixing, reaching their final composition after 5 weeks. a,b) ^29^Si NMR spectra, c) ^1^H NMR spectrum, and d) ^13^C NMR spectrum of precursor solution B directly after mixing, and after 1, 2, 3, and 5 weeks of aging at room temperature. e) Molecular structures of TEOS, TMSPMA, and EMITFSI. □: ethanol (used as solvent and formed by the hydrolysis of TEOS). △: methanol (formed by the hydrolysis of TMSPMA). x: exchangeable protons of ethanol, methanol, and water.

The chemical properties of precursor solution B were followed over the course of time using ^29^Si, ^1^H, and ^13^C nuclear magnetic resonance (NMR) spectroscopy (**Figure** **2**). Directly after mixing, the ^29^Si NMR spectrum contained two distinct peaks at −40 and at −80 ppm, attributed to the Si atoms of TMSPMA and TEOS, respectively (Figure 2a). The hydrolysis and condensation reactions of TMSPMA occur significantly faster than those of TEOS, as can be inferred from the rapid decrease in its peak intensity with time. After 2 weeks, the TMSPMA signal nearly disappeared, whereas there was still a significant TEOS signal. A closer inspection of the same spectra allows to determine which condensed species are formed (Figure 2b). In ^29^Si NMR spectroscopy, “T” designates trifunctional units (i.e., TMSPMA). The codes T^1^, T^2^, and T^3^ signify that these units are condensed with one, two, and three other alkoxysilanes, respectively (as shown in Figure S1, Supporting Information).^[^ ^25^ ^]^ Similarly, the codes Q^1^, Q^2^, Q^3^, and Q^4^ are used for TEOS units that are condensed with one, two, three, and four other alkoxysilanes, respectively. Already immediately after mixing, there is a significant T^1^ signal, indicating that TMSPMA rapidly undergoes hydrolysis and condensation. Whereas also Q^1^ structures are already formed at this timepoint, their signal intensity is much smaller. This indicates that the beginning of the aging reaction mainly involves the self‐condensation of TMSPMA molecules, with only a small amount of TEOS being incorporated. After 1 week of aging, a significant amount of T^3^ structures (i.e., fully condensed TMSPMA) has been formed. In contrast, the presence of Q^2^, Q^3^, and Q^4^ structures is difficult to verify due to the presence of a broad background band from −90 to −130 ppm from the glass of the NMR tube. Still, it is clear that the condensation of T structures occurs significantly faster than the condensation of Q structures. The formation of more condensed Q structures becomes more evident with further aging. After 5 weeks, the TMSPMA and TEOS molecules have mostly been converted into T^3^ and Q^4^ structures, respectively, with only a minor amount of less condensed structures present.

The use of TEOS as sole silica precursor molecule leads to a spontaneous solidification process.^[^ ^23^ ^]^ This implies that the fast hydrolysis and condensation kinetics of TMSPMA are essential to avoid the self‐condensation of TEOS. The condensation of TMSPMA and TEOS results in the formation of solubilized condensed structures. This can be inferred from the indefinite stability of precursor solutions A and B, where no light scattering can be observed with the naked eye or through the Tyndall effect (Figure S2, Supporting Information).

In the ^1^H and ^13^C NMR spectra (Figure 2c,d; Figure S3, Supporting Information), the signals of the alkoxy group atoms slowly disappear over time. This occurs faster for TMSPMA than for TEOS, corroborating the observation from the ^29^Si spectra. Methanol is formed throughout the aging process through the hydrolysis of the TMSPMA methoxy groups. The signals that relate to the ILE (i.e., those of EMITFSI) remain unchanged throughout the observed 5 weeks, indicating that the ILE remains stable during this time period. The signals that relate to the organic chain of TMSPMA broaden over the course of time. This indicates that the structure becomes less mobile, which implies that these molecules are incorporated into larger structures, i.e., organically modified silica structures are formed. This was also observed in the ^29^Si spectra. The changes in ^29^Si, ^1^H, and ^13^C spectra in the period between 3 and 5 weeks are minute. Therefore, we presume that the hydrolysis and condensation reactions are nearly complete after 5 weeks, and that the organically modified silica clusters have reached their final composition after this time period.

#### 2.1.2 Solidification

**Figure 3:**
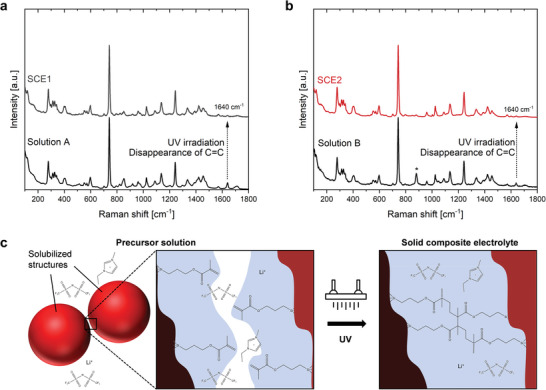
The solidification process is induced by the linking of the vinylidene moieties of the organically modified silica structures. a) Raman spectra of precursor solution A and SCE1 before drying. b) Raman spectra of precursor solution B and SCE2 before drying. The presence of the solvent in precursor solution B is indicated with an asterisk. c) Scheme depicting the SCE solidification process.

Based on the NMR results, the precursor solutions have adequately aged after a period of five weeks. Therefore, SCEs were prepared from the precursor solutions after this aging time. Precursor solutions A and B were used to prepare a set of four SCEs. SCE1 and SCE2 were prepared by the addition of 5 mg mL^−1^ 2,2‐dimethoxy‐2‐phenylacetophenone (DMPA) as photoinitiator to precursor solutions A and B, respectively, followed by oxygen stripping and 1 h of UV irradiation. Whereas the solutions were irradiated for 1 h, the formation of monoliths could already be observed after a period of ≈5 min. The solidification mechanism of both precursor solutions was studied by comparing their Raman spectra before and after the solidification step (**Figure** **3**). Although the spectra are dominated by the ILE (Figure S4, Supporting Information),^[^ ^26^, ^27^, ^28^, ^29^ ^]^ the solidification process is coupled with the disappearance of the peak at 1640 cm^−1^. This peak relates to the presence of the vinylidene (C═CH_2_) moiety originating from TMSPMA.^[^ ^30^, ^31^ ^]^ This implies that the solidification process is induced by the crosslinking of separate silica structures through the polymerizable moieties present on the organosilane chains (Figure 3c).

Two additional SCEs were synthesized from solution B. SCE3 was prepared by adding extra LiTFSI/EMITFSI (1:3 molar ratio) to precursor solution B, reaching a [EMITFSI]:[Si] ratio of 4. SCE4 was prepared by similarly adding a LiFSI/EMIFSI (1:3 molar ratio) solution to solution B, producing a system that contains FSI^−^ as well as TFSI^−^ anions. The solidification procedure occurred as mentioned above, and the presence of a higher amount of ILE did not inhibit solidification (Figure S5, Supporting Information). The characteristics of the four SCEs are summarized in **Table** **2**.

**Table 2:** Used precursor solution, [EMITFSI]:[Si] ratio, [EMIFSI]:[Si] ratio, organic content, and ionic conductivity at 20 °C of the SCEs presented in this article.

Sample	Precursor solution	[EMITFSI]:[Si]	[EMIFSI]:[Si]	Organic wt% in solid matrix	Ionic conductivity at 20 °C [mS cm^−1^]
SCE1	A	1.5	0	71	1.0
SCE2	B	1.5	0	53	1.4
SCE3	B	4	0	53	2.6
SCE4	B	1.5	2.5	53	2.7

#### 2.1.3 Drying

After the solidification process, the SCEs still contain water and solvent, which must be removed before the SCEs can be incorporated in a battery cell. Therefore, the “wet” SCEs were stored in a dry room (dew point < −45 °C) and their weight was monitored until a steady–state was reached. Over the course of the drying process, the SCE monoliths underwent a small extent of shrinkage.

**Figure 4:**
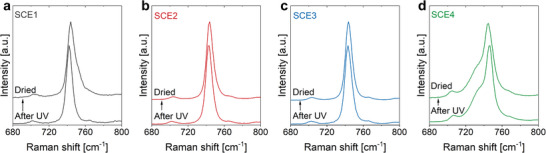
The drying process leads to molecular ordering of the ILE on the matrix’ silica surface, which prevents Li^+^‐TFSI^−^ association in SCE4, SCE3, and SCE2, whereas this is not the case for SCE1. Raman spectra before and after drying of a) SCE1, b) SCE2, c) SCE3, and d) SCE4.

The presence (and removal) of water can have a pronounced effect on the molecular interactions within the SCEs, and more specifically, on the interaction of the lithium ions with the anions. In Raman spectroscopy, the collective expansion/contraction modes of the TFSI^−^ anion influence the position/shape of the peak ≈740 cm^−1^. This peak therefore provides information on the anion's conformation and coordination with lithium ions.^[^ ^32^ ^]^ A similar peak shows up ≈730 cm^−1^ for the FSI^−^ anion. Raman spectroscopy (along with attenuated total reflectance Fourier‐transform infrared (ATR‐FTIR) spectroscopy)^[^ ^19^, ^33^, ^34^ ^]^ was therefore conducted on the SCEs to assess the lithium ion coordination environment (**Figure** **4**; Figures S6 and S7, Supporting Information). In the Raman spectra of “wet” SCE1, SCE2, and SCE3 (which all contain TFSI^−^ as sole anion), the peak at 743 cm^−1^ is narrow, as is the case for EMITFSI (Figure S4, Supporting Information). This contrasts with the LiTFSI/EMITFSI solution (Figure S4, Supporting Information), where significant peak broadening indicates the formation of complexes such as [Li(TFSI)_2_]^−^.^[^ ^34^ ^]^ The narrow peaks in the “wet” SCEs indicate that such complexes are not present, i.e., that there is little interaction between Li^+^ and TFSI^−^. For SCE1 (Figure 4a), the drying process results in peak broadening, which indicates that TFSI^−^ strongly associates with lithium ions in this sample.^[^ ^34^, ^35^ ^]^ For SCE2 (Figure 4b), there is little broadening and for SCE3 (Figure 4c), the drying process does not lead to any observable peak broadening. Li^+^ does not strongly associate with TFSI^−^ in SCE3. Therefore, it can be concluded that the interaction with the solid matrix mitigates the association between Li^+^ and TFSI^−^. A similar observation was made for an ILE enclosed in a silica matrix by Chen et al.,^[^ ^23^ ^]^ who attributed this effect to the association of TFSI^−^ with the hydroxylated silica surface through an adsorbed ice layer that is not electrochemically active. Considering that this effect relies on ILE molecular ordering on the silica surface, we ascribe the decrease in Li^+^‐TFSI^−^ association from SCE1 to SCE2 and SCE3 to the increase in silica content (from SCE1 to SCE2 and SCE3) and to a potential increase in ILE/silica interfacial area. SCE4 contains both TFSI^−^ and FSI^−^ anions, and the Raman spectra before and after drying (Figure 4d) are virtually identical. Here, the presence of two anion types, in addition to the hybrid solid matrix, makes the resolution of the lithium ion solvation structure practically impossible.^[^ ^7^ ^]^ Still, the absence of peak broadening indicates that the lithium/anion interaction is limited.

### 2.2 Thermal Behavior

The thermal stability of the dried SCEs is dominated by the incorporated ILE, as determined by thermogravimetric analysis (TGA) under dry air atmosphere (Figure S8, Supporting Information).^[^ ^36^, ^37^ ^]^ SCE1, SCE2, and SCE3 are stable up to ≈300 °C, irrespective of the ILE:matrix ratio. SCE4 starts to degrade at ≈180 °C, concurrent with the lower thermal stability of LiFSI/EMIFSI.^[^ ^7^ ^]^ The decomposition of the ILE is responsible for a large mass loss, which is complete at ≈500 °C. Subsequently, a small amount of mass loss can be observed for the SCEs, relating to the degradation of the organic fraction of the matrix. In differential scanning calorimetry (DSC, Figure S9, Supporting Information), all SCEs have a glass transition temperature of ≈ −76 °C. The absence of other outspoken thermal phenomena within the investigated temperature range indicates that the SCEs behave as homogeneous systems.

### 2.3 Ionic Conductivity

**Figure 5:**
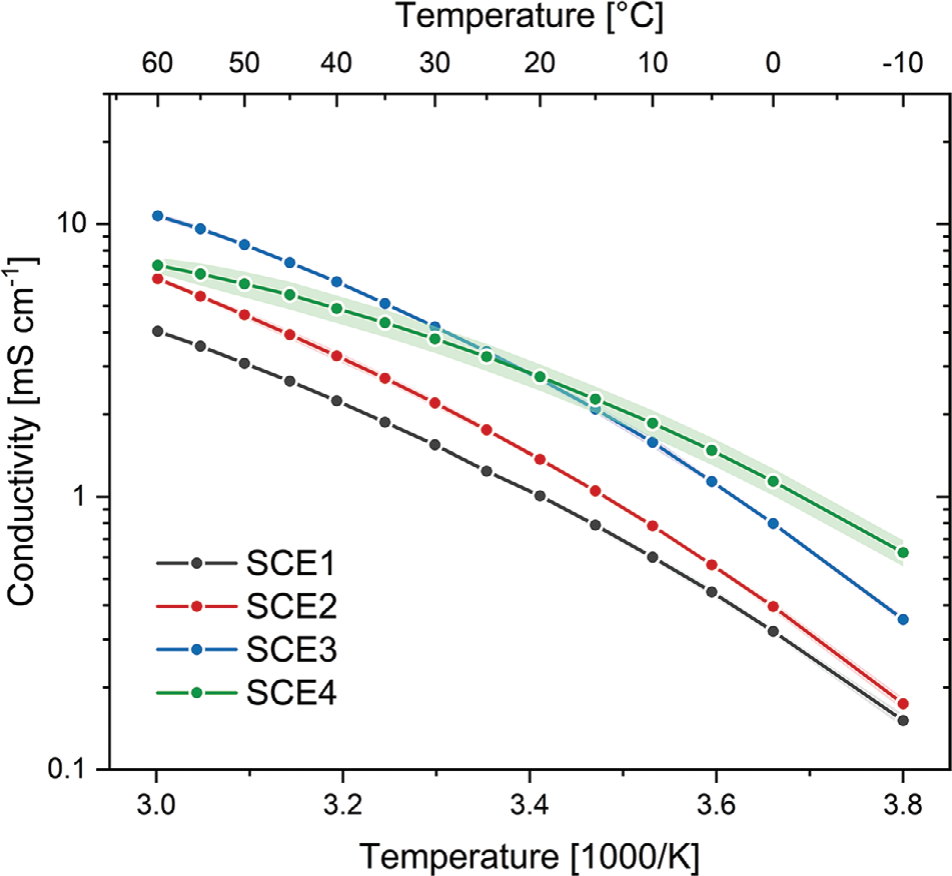
Arrhenius plot of the ionic conductivity of the SCEs. Data points, which are the average of three separate measurements, are represented by dots, whereas the lines are only meant to guide the eye. The shaded area represents the standard deviation for every sample, calculated from three separate measurements.

The conductivity of the four dried SCEs was measured in function of temperature (**Figure** **5**). At 20 °C, their conductivity values are 1.0 mS cm^−1^ (SCE1), 1.4 mS cm^−1^ (SCE2), 2.6 mS cm^−1^ (SCE3), and 2.7 mS cm^−1^ (SCE4). The increase in conductivity from SCE1 to SCE3 can be partially explained by the higher ILE content in the same order. SCE2 has a higher ILE content than SCE1 because of the lower molecular weight (i.e., lower organic content) of the solid matrix. SCE3 has a higher ILE content than SCE2 because of its higher ILE:matrix ratio (Table 2). However, because the conductivity of SCE3 was systematically higher than that of the neat ILE at 20 °C and below (Figure S10, Supporting Information), there must be other factors at play which enhance the conductivity of the SCEs compared to the ILEs. As described by Chen et al.,^[^ ^23^ ^]^ a solid‐like surface water or ice layer (which itself is not electrochemically active) on the silica surface of similar materials can promote the conduction of lithium ions. After all, the molecular ordering of the ILE ions on the silica surface results in the dissociation of Li^+^‐TFSI^−^ interaction, increasing the mobility of Li^+^ and therefore also increasing the ionic conductivity. In our case, the Li^+^‐TFSI^−^ dissociation was shown to increase from SCE1 to SCE2 and SCE3 (Figure 4). The molecular ordering on the interface may also be influenced by the temperature, with more ordering at lower temperatures and less ordering at higher temperatures. This may explain why the conductivity enhancement is more outspoken at lower temperatures, resulting in SCE3 having a higher ionic conductivity than the ILE. SCE4 contains the same molar amount of ILE as SCE3, but with a mixture of TFSI^−^ and FSI^−^ anions (Table 2). In liquid systems, the replacement of TFSI^−^ with FSI^−^ leads to an overall increase in conductivity because of the decrease in viscosity, itself a consequence of the smaller anion size and weaker cation/anion interaction.^[^ ^7^, ^38^, ^39^ ^]^ At room temperature, the ionic conductivity values of SCE3 and SCE4 are nearly identical, further indicating that the Li^+^‐TFSI^−^ association has weakened. At lower temperatures, SCE4 has a higher ionic conductivity than SCE3, while the roles are reversed at higher temperatures (Figure 5). This is a mark of the complex nature of the chemical interactions governing the ionic conductivity in the SCEs. For all SCEs, the variation of the ionic conductivity with temperature could be fit to the Vogel−Tammann−Fulcher (VTF) equation (Table S1, Supporting Information).^[^ ^40^, ^41^, ^42^ ^]^ Because of their superior ionic conductivity, SCE3 and SCE4 were selected for further electrochemical characterization.

### 2.4 Electrode Compatibility

**Figure 6:**
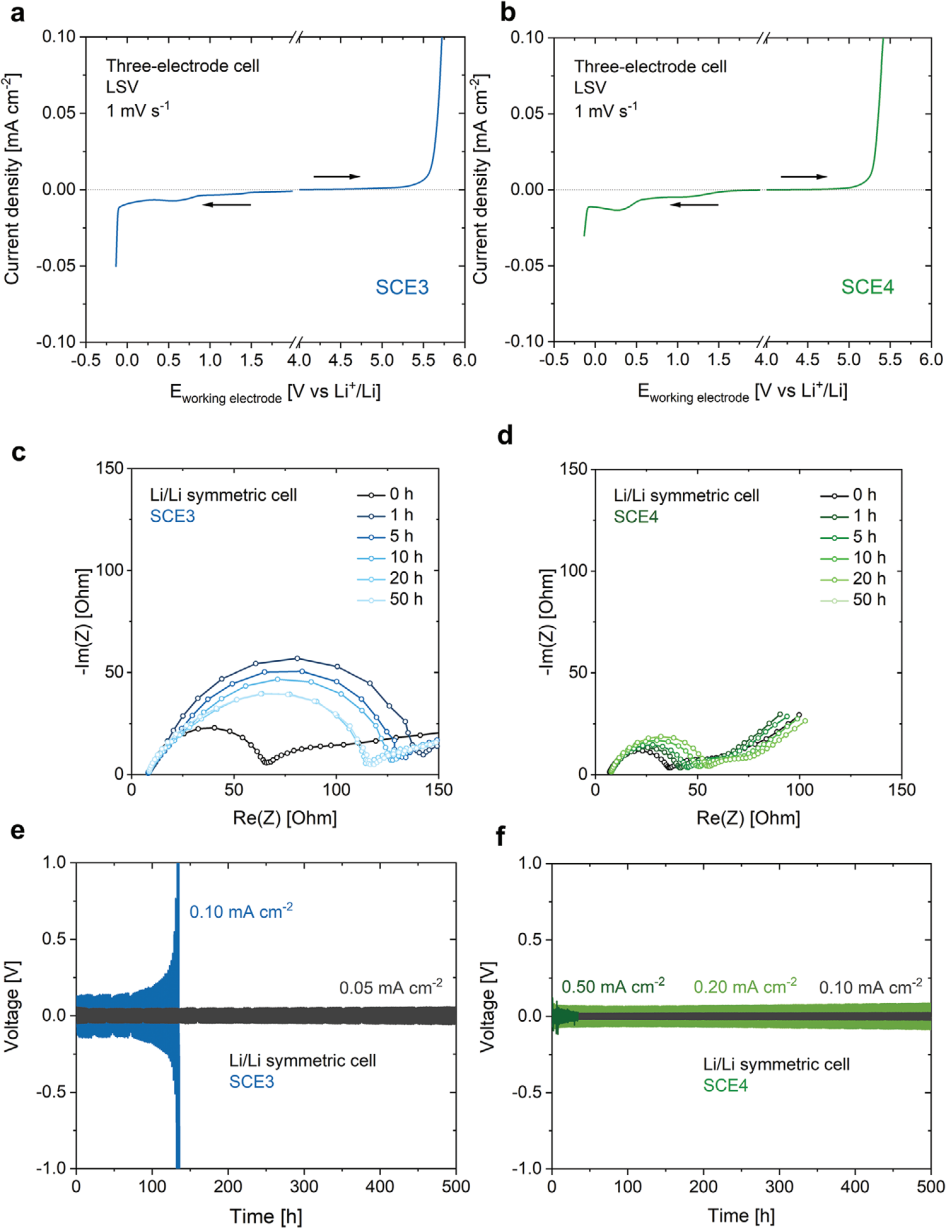
The SCEs have a broad electrochemical stability window, and SCE4 forms a stable degradation layer with excellent passivating properties on the interface with lithium metal. Anodic and cathodic LSV of a) SCE3 and b) SCE4. The measurements were performed in a three‐electrode cell at room temperature at a scan rate of 1 mV s^−1^. Nyquist plot representation of the impedance spectra of symmetric Li/Li cells containing c) SCE3 and d) SCE4 as electrolyte. The impedance spectra were recorded over a frequency range of 1 MHz–10 mHz and subject to a potential amplitude of 10 mV. Galvanostatic cycling of a symmetric e) Li/SCE3/Li and f) Li/SCE4/Li cell at various current densities (1 h per half cycle).

The anodic stability limit of SCE3 was investigated by impregnating it in a glass fiber separator and performing linear sweep voltammetry (LSV) on a three‐electrode cell with a stainless steel plate as the working electrode, a piece of lithium foil as the counter electrode, and lithium as the reference electrode (**Figure** **6**a). This revealed that SCE3 has an anodic stability limit >5 V versus Li^+^/Li, and therefore potentially is compatible with high‐voltage positive electrode materials. Still, one should keep in mind that the anodic stability thresholds established with this technique are somewhat arbitrary.^[^ ^43^ ^]^ A cathodic LSV experiment on a similar cell showed the reduction of the TFSI^−^ ions^[^ ^44^ ^]^ at ≈1.2 V versus Li^+^/Li and of the EMI^+^ ions^[^ ^45^ ^]^ at ≈0.7 V versus Li^+^/Li. Lithium plating occurs <0 V versus Li^+^/Li. The passivating properties of the Li/SCE3 degradation layers were studied by assembling symmetric Li/SCE3/Li cells (where the SCE was again impregnated in a glass fiber separator) and performing electrochemical impedance spectroscopy after certain time intervals (Figure 6c). Directly after cell assembly, the cells have a low charge transfer resistance (*R*_ct_), which increases significantly after 1 h. The *R*_ct_ then gradually decreases with time until it reaches steady state after 20 h (i.e., *R*_ct_ is equal after 20 h and after 50 h). This implies that the degradation layers are quickly formed after cell assembly, and that they gradually become more stable and less resistive to the transfer of lithium ions. Similar symmetric cells were subjected to galvanostatic cycling (Figure 6e; Figure S11, Supporting Information).^[^ ^46^ ^]^ The cells showed stable lithium plating and stripping for at least 500 h with only a slight increase in polarization at a current density of 0.05 mA cm^−2^ (capacity 0.05 mAh cm^−2^ per half cycle). At 0.10 mA cm^−2^ (capacity 0.10 mAh cm^−2^ per half cycle), the polarization increased throughout the cycling process until the cell failed after ≈140 h. This suggests that lithium plating and stripping is non‐uniform, and that the passivating SEI formed during this process cannot ensure long‐term stable cycling of lithium electrodes at this current density.^[^ ^47^, ^48^ ^]^

The same set of experiments was applied to SCE4. Its anodic stability limit is somewhat lower than that of SCE3, as was previously reported for mixtures of TFSI^−^ and FSI^−^ (Figure 6b).^[^ ^7^ ^]^ Still, the stability limit appears to be higher than 5 V versus Li^+^/Li, implying that also this SCE may be compatible with state‐of‐the‐art positive electrodes. In the cathodic scan, the plating current is lower than was the case for SCE3. As the ionic conductivity is similar in both cases, this may be caused by the formation of more stable degradation layers on the Li/SCE4 interface. Alternatively, the lithium plating at the working electrode may be limited by the lithium stripping process at the counter electrode. The passivation layers are less resistive to the flow of ions than was the case for Li/SCE3/Li cells, as indicated by the considerably lower *R*_ct_ values for Li/SCE4/Li cells (Figure 6d). When these were subjected to galvanostatic cycling, they showed stable operation at a current density of 0.20 mA cm^−2^ (0.20 mAh cm^−2^ per half cycle, Figure 6f), i.e., double as high as Li/SCE3/Li cells. However, the cell quickly shorted at a current density of 0.50 mA cm^−2^.

### 2.5 Electrochemical Performance

**Figure 7:**
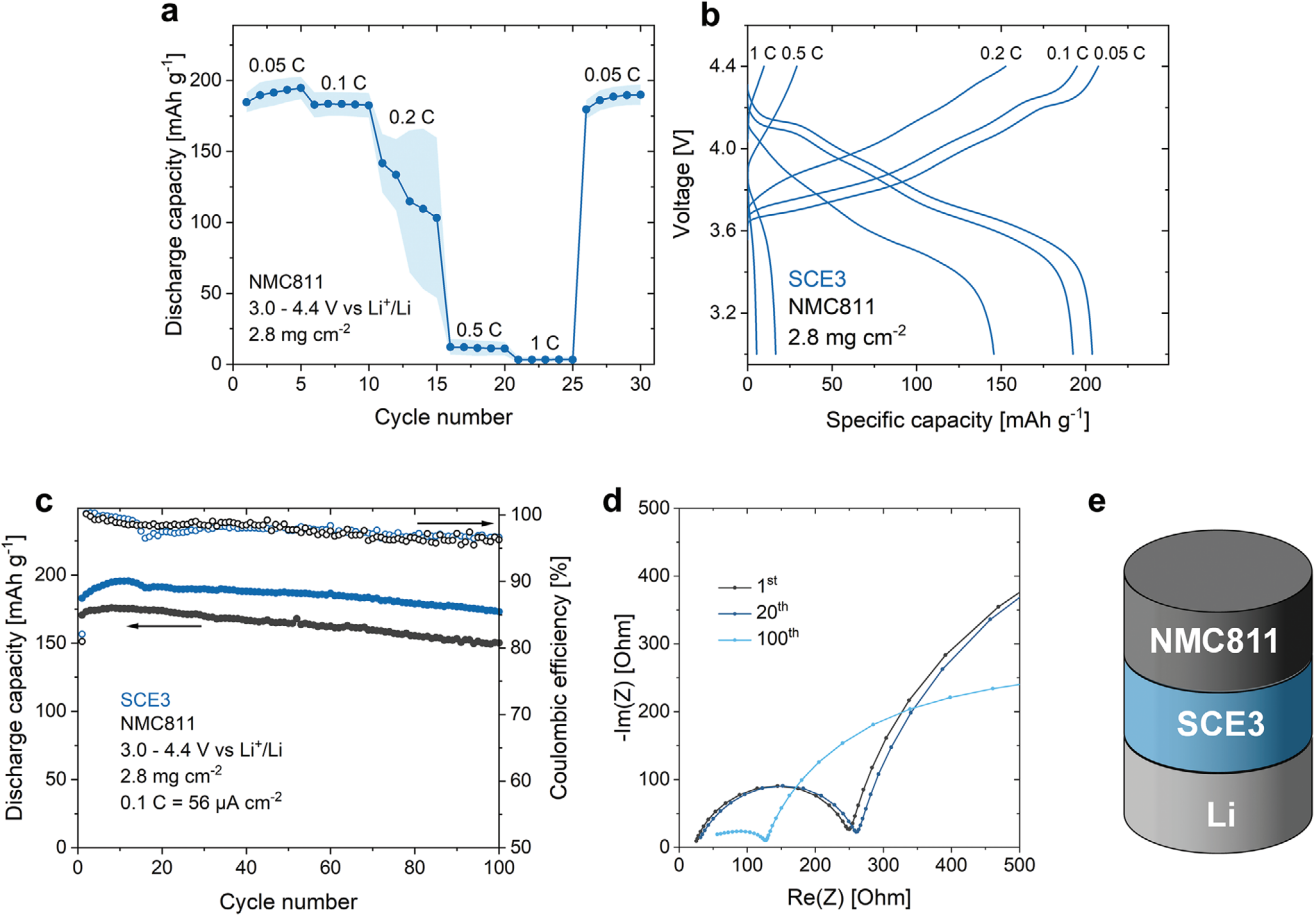
SCE3 has an excellent compatibility with NMC811, but the stability of the lithium plating/stripping process is limited. a) Discharge capacity of NMC811/SCE3/Li coin cells at rates from 0.05 C to 1 C. The shaded areas indicate the standard deviation according to three separate measurements. b) Related galvanostatic charge/discharge curves. c) Capacity retention and coulombic efficiency of two NMC811/SCE3/Li coin cells at 0.1 C. d) Nyquist plot representation of the impedance spectra of NMC811/SCE3/Li coin cells after 1, 20, and 100 cycles at 0.1 C. e) Schematic representation of the investigated cells. All tests were conducted at room temperature between 3.0 and 4.4 V versus Li^+^/Li. The average NMC811 mass loading was 2.8 mg cm^−2^, equivalent to 0.56 mAh cm^−2^. As such, 1 C corresponds to 0.56 mA cm^−2^.

SCE3 was incorporated into NMC811/Li half‐cell coin cells by impregnating the precursor solution into a stack of an electrode punch and a glass fiber separator, followed by UV irradiation and drying in a dry room (until no more mass loss could be observed). As indicated by scanning electron microscopy (SEM, Figures S12–S14, Supporting Information), this ensured the impregnation of the SCE into the electrode pores. After assembly, the cells were cycled within the window of 3–4.4 V versus Li^+^/Li, assuming a theoretical capacity of 200 mAh g^−1^. At 0.05 and 0.1 C, the cells reached an average discharge capacity of 195 and 183 mAh g^−1^, respectively (**Figure** **7**a). These high capacities, along with the low polarization observed in the galvanostatic charge/discharge curves (Figure 7b), indicate that SCE3 is electrochemically compatible with NMC811 and with lithium metal. However, at 0.2 C, the cells experience a pronounced capacity decay along with an increase in polarization. At an active material loading of 2.8 mg cm^−2^, 0.2 C corresponds to 0.112 mA cm^−2^, which is higher than SCE3's critical current density for lithium plating/stripping (Figure 6e). We therefore attribute the fast capacity decay to instabilities at the SCE3/Li interface.

The stability of NMC811/SCE3/Li cells was studied below the critical current density for lithium plating/stripping, i.e., at 0.1 C. Two cells were assembled to ensure the reproducibility of the results (Figure 7c). The first cell had an initial discharge capacity of 171 mAh g^−1^ and a capacity retention of 88.0% after 100 cycles. For the second cell, these values were 183 mAh g^−1^ and 94.6%. We attribute the difference between the cells to slight variations in stack pressure, which cannot be accurately controlled in coin cells.^[^ ^49^ ^]^ For both cells, the coulombic efficiency is higher than 99% during the initial cycles, but gradually diminishes to ≈96% in the 100th cycle. This indicates that the plated lithium is continuously degraded by SCE3. In other words, the formed degradation layers cannot provide the necessary passivation of the lithium electrode. This is in line with previous reports, where the degradation of the TFSI^−^ anion on lithium metal led to the formation of a poorly passivating SEI layer, which leads to instability of the lithium plating/stripping process.^[^ ^50^ ^]^ Still, the *R*_ct_ decreases over the course of cycling (Figure 7d), which might find its origin in an improving interfacial contact. This could, however, not be confirmed or denied experimentally (Figure S15, Supporting Information).

**Figure 8:**
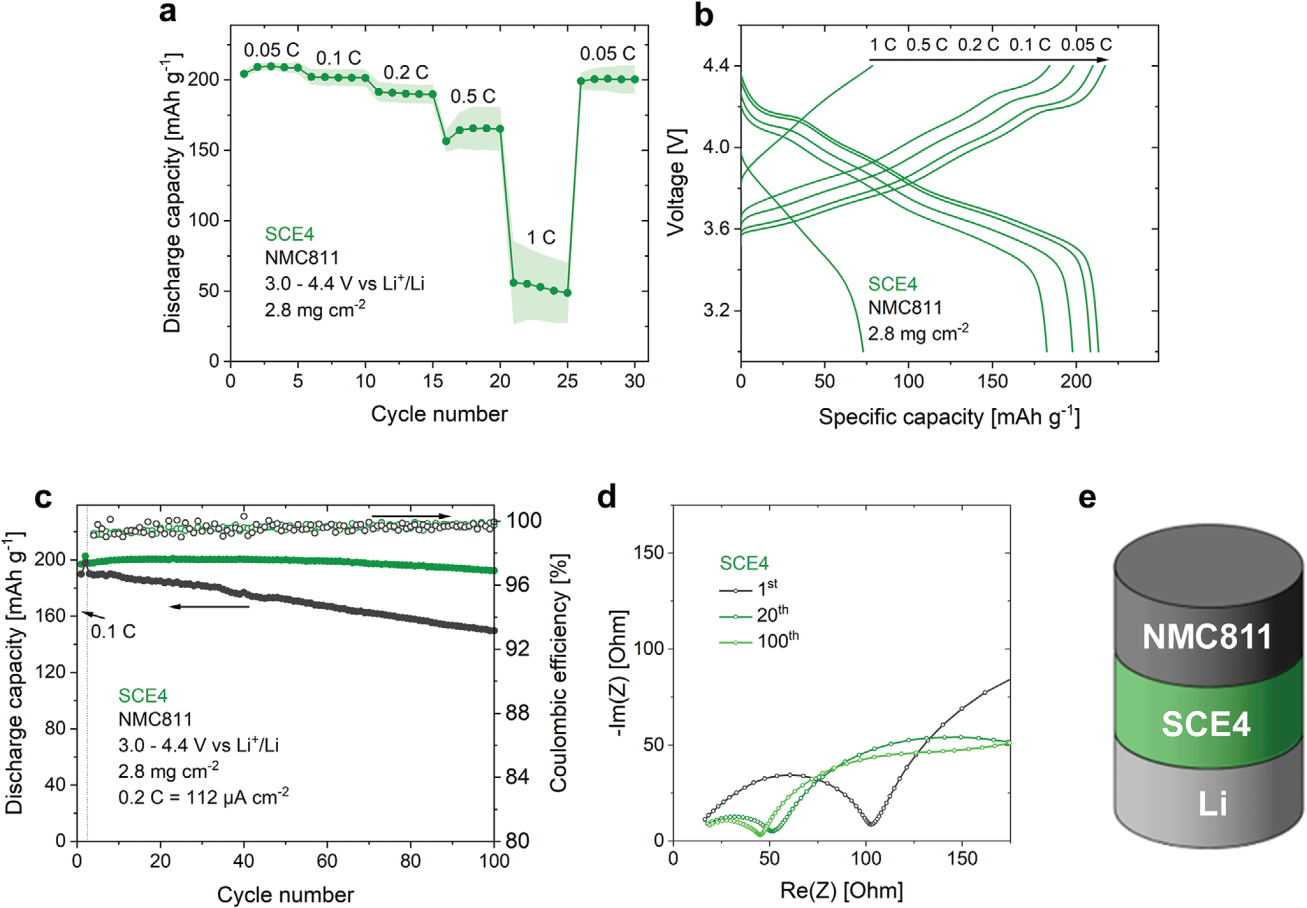
SCE4 is compatible with NMC811 as well as with lithium metal. a) Discharge capacity of NMC811/SCE4/Li coin cells at rates from 0.05 C to 1 C. The shaded areas indicate the standard deviation according to three separate measurements. b) Related galvanostatic charge/discharge curves. c) Capacity retention and coulombic efficiency of two NMC811/SCE4/Li coin cells at 0.2 C. d) Nyquist plot representation of the impedance spectra of NMC811/SCE4/Li coin cells after 1, 20, and 100 cycles at 0.2 C. e) Schematic representation of the investigated cells. All tests were conducted at room temperature between 3.0 and 4.4 V versus Li^+^/Li. The average NMC811 mass loading was 2.8 mg cm^−2^, equivalent to 0.56 mAh cm^−2^. As such, 1 C corresponds to 0.56 mA cm^−2^.

The combination of TFSI^−^ and FSI^−^ as anions, as in SCE4, was reported to have a positive influence on the reversibility of the lithium plating/stripping process.^[^ ^51^, ^52^, ^53^, ^54^ ^]^ SCE4 was incorporated into NMC811/Li half‐cell coin cells using the procedure described above. The cells have a higher discharge capacity than the SCE3‐based cells, reaching 209, 201, and 190 mAh g^−1^ at 0.05 C, 0.1 C, and 0.2 C, respectively (**Figure** **8**a,b). At higher current rates, the discharge capacities become more irreproducible and unstable, in line with SCE4's critical current density (Figure 6f). Two cells were subjected to a cycle stability test at 0.2 C (preceded by two activation cycles at 0.1 C) (Figure 8c). One cell has an initial capacity (3rd cycle, 0.2 C) of 198 mAh g^−1^ and retains 97.4% in the 100th cycle. For the second cell, these values are 190 mAh g^−1^ and 78.7%. In contrast to SCE3, the coulombic efficiency remains consistently >99% throughout the cycling process, indicating that SCE4 has an improved compatibility with lithium metal, leading to the formation of a stable, passivating interface. The electrochemical stability of the cell is further corroborated by the decreasing *R*_ct_ over the course of cycling (Figure 8d).

**Figure 9:**
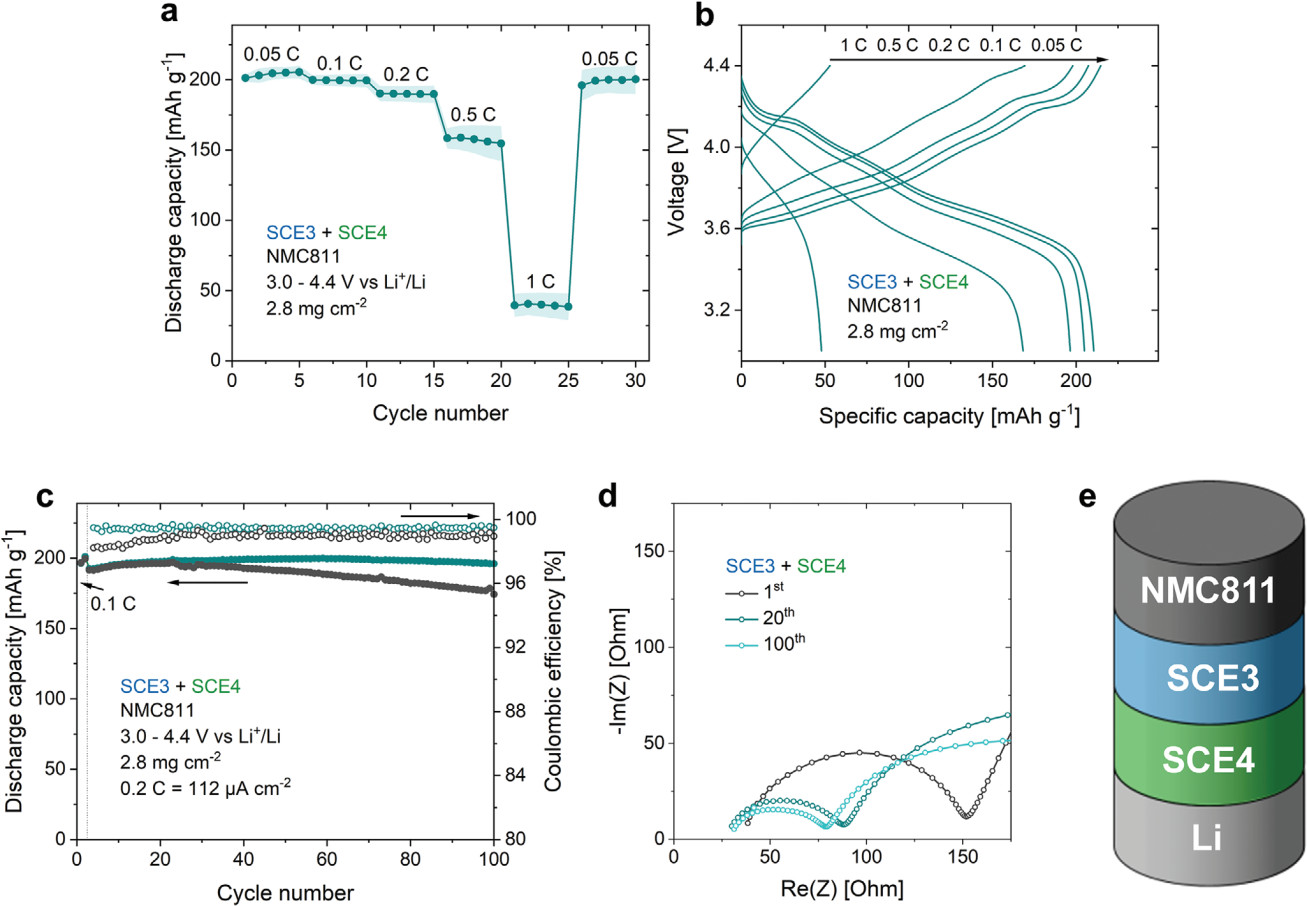
The electrochemical reproducibility can be further improved by using SCE3 as catholyte and SCE4 as anolyte. a) Discharge capacity of NMC811/SCE3/SCE4/Li coin cells at rates from 0.05 C to 1 C. The shaded areas indicate the standard deviation according to three separate measurements. b) Related galvanostatic charge/discharge curves. c) Capacity retention and coulombic efficiency of two NMC811/SCE3/SCE4/Li coin cells at 0.2 C. d) Nyquist plot representation of the impedance spectra of NMC811/SCE3/SCE4/Li coin cells after 1, 20, and 100 cycles at 0.2 C. e) Schematic representation of the investigated cells. All tests were conducted at room temperature between 3.0 and 4.4 V versus Li^+^/Li. The average NMC811 mass loading was 2.8 mg cm^−2^, equivalent to 0.56 mAh cm^−2^. As such, 1 C corresponds to 0.56 mA cm^−2^.

With its higher anodic stability, SCE3 has a better stability toward NMC811 than SCE4. On the other hand, SCE4 has an improved stability toward lithium metal. This implies that further improvements in electrochemical stability and reproducibility may be achieved by using a combination of SCE3 as catholyte (in contact with the positive electrode) and SCE4 as anolyte (in contact with lithium metal). Such cells were assembled by first impregnating the porous positive electrode with SCE3 precursor solution and subjecting it to UV light, followed by drying. The SCE4 precursor solution was impregnated in a thin glass fiber separator, and the battery assembly proceeded as explained before. These cells have a very similar rate performance to the SCE4‐based cells, reaching 209, 199, and 190 mAh g^−1^ at 0.05 C, 0.1 C, and 0.2 C respectively (**Figure** **9**a,b). Again, the capacity at higher currents is limited by the lithium negative electrode, reaching 158 mAh g^−1^ at 0.5 C and 40 mAh g^−1^ at 1 C. The improved reproducibility is apparent in the cycle stability tests, where both cells have an initial discharge capacity of 192 mAh g^−1^ (third cycle, 0.2 C), and retain 100% and 91.1% of this capacity in the 100th cycle (Figure 9c). Similar to the NMC811/SCE4/Li cells, the NMC811/SCE3/SCE4/Li cells show a decreasing *R*_ct_ with cycle number (Figure 9d).

## 3 Conclusion

We describe a novel type of hybrid SCE as solid electrolyte in lithium batteries, which combines functional properties with electrode compatibility and good manufacturability. The acid‐free precursor solution can be readily impregnated into porous electrodes without damaging them and can then be instantaneously solidified by irradiation with UV light. In the resulting monolith, an ILE is immobilized within a solid matrix. This induces molecular ordering on the ILE/silica interface, which in turn increases the mobility of lithium ions. This leads to an increase in ionic conductivity (up to 2.7 mS cm^−1^ at room temperature, higher than the neat ILE). Using a LiTFSI/EMITFSI solution as ILE, the SCE has an anodic stability limit >5 V Li^+^/Li, and is compatible with NMC811‐based electrodes. However, its poor stability toward lithium metal limits the rate performance of NMC811/Li cells. Using an ILE consisting of a mixture of LiFSI, LiTFSI, EMIFSI, and EMITFSI slightly lowers the corresponding SCE's anodic stability, but allows the formation of robust, conductive degradation layers on the SCE/Li interface. Using the former SCE as catholyte, and the latter as anolyte, NMC811/Li cells have a high electrochemical stability, retaining 91–100% of their initial capacity after 100 cycles (0.2 C). The combination of functional properties, electrode compatibility, and manufacturability shows that this type of SCE is a potential candidate for the further development of solid‐state lithium metal battery technology, provided that its compatibility with lithium metal can be further improved.

## 4 Experimental Section

### Precursor Solutions

Precursor solutions A and B were prepared by mixing LiTFSI (Solvionic, 99.9%), EMITFSI (Solvionic, 99.9%), TEOS (Alfa Aesar, 98%), TMSPMA (Alfa Aesar, 97%), milliQ water, and solvent in the ratios described in Table 1 (performed in ambient conditions). The solvent for precursor solution A was 1‐methoxy‐2‐propanol (PGME, Alfa Aesar, 99+%). The solvent for precursor solution B was ethanol (VWR, ≥99.8%).

### Monolith Solidification

SCE monoliths were prepared from precursor solutions that had aged at least 5 weeks. The relationships between precursor solutions and SCEs are detailed in Table 2. For SCE3, additional LiTFSI and EMITFSI were added to precursor solution B. For SCE4, additional LiFSI (Solvionic, 99.9%) and EMIFSI (Solvionic, 99.9%) were added to precursor solution B. A concentration of 5 mg mL^−1^ photoinitiator DMPA (Acros organics, 99%) was dissolved in the desired amount of precursor solution. Then, dissolved O_2_ was stripped from the precursor solutions by N_2_ bubbling (30 min). Subsequently, a volume of 400 µL precursor solution was poured in a mold (19 mm diameter) and directly irradiated with a Hg UV lamp (UV‐A, 1 h). Thereafter, the water and solvent were removed from the monoliths by placing them in a dry room at room temperature (dew point < −45 °C) until their mass remained stable, indicating that all volatiles were removed.

### Physicochemical Characterization

NMR spectroscopy was performed on precursor solution B after certain aging times. The peak attributions were performed by iterative addition of the solution's components. NMR spectra were acquired with a 5 mm Royal HFX probe at RT on a Jeol ECZ400R spectrometer at 399.782, 100.525, and 79.425 MHz for ^1^H, ^13^C, and ^29^Si spectra, respectively. Acquisition parameters used were i) for ^1^H NMR: a spectral width of 6 kHz (15 ppm), a 90° pulse length of 6.6 µs, an acquisition time of 2.2 s, a recycle delay time of 15 s and 64 accumulations; ii) for ^13^C NMR: a spectral width of 30 kHz (300 ppm), a 90° pulse length of 10.9 µs, an acquisition time of 0.9 s, a recycle delay time of 6 s and ≈600 accumulations; and iii) for ^29^Si NMR: a spectral width of 23.8 kHz (300 ppm), a 90° pulse length of 12.8 µs, an acquisition of 1.1 s, a recycle delay time of 10 s and ≈20 000 accumulations. Raman spectra were collected (Renishaw InVia Qontor Confocal Raman Microscope, 785 nm excitation wavelength (100 mW)) at room temperature. The Raman shift scale was calibrated using silicon. The dried SCEs were placed in an airtight cell before the measurement, allowing data acquisition without exposure to ambient moisture. The measurements were performed in a continuous scan mode (exposure time of 10 s, 100% laser power, and a 1200 l mm^−1^ grating). Cosmic rays were removed when present. For ATR‐FTIR spectroscopy, a Perkin Elmer Frontier FTIR spectrometer, fitted with a Pike MIRacle single reflection ATR accessory, was utilized (32 scans, 4000–600 cm^−^¹ scan range, 4 cm^−^¹ resolution). TGA (TA instruments Q600) was performed by heating 6−8 mg of the samples at 10 °C min^−1^ to 600 °C under dry air flow. A TA Instruments Q200 differential scanning calorimeter was used to conduct DSC. The SCEs were placed in aluminum hermetic pans and analyzed over three cycles (−90–50 °C, 10 °C min^−1^, nitrogen flow of 50 mL min^−1^). SEM images of (impregnated) electrodes were acquired on a TESCAN VEGA (secondary electron (SE) mode for topography) and on a Zeiss 450 FEG‐SEM with Gemini 2 column (backscattered electron (BSE) mode for Z contrast).

### Electrochemical Characterization

The conductivity of the neat ILE was measured using a Mettler Toledo FiveEasy Plus conductometer, with the samples placed in a thermostatic bath. The ionic conductivity of the SCEs at different temperatures was determined in the dry room by performing potentiostatic electrochemical impedance spectroscopy (PEIS, Bio‐Logic SP‐300, 7 MHz–100 mHz, 10 mV amplitude) on monoliths (thickness ≈1 mm) between two stainless steel plates. The set‐up was placed in a Binder oven and the temperature was controlled between −10 and 60 °C. Li/SCE/Li cells were subjected to PEIS (Bio‐Logic VMP3, 1 MHz–10 mHz, 10 mV amplitude). Here, 100 µL precursor solution was impregnated into a glass fiber separator (EL‐CELL, 19 mm diameter and 0.26 mm thickness) and subsequently treated similarly to the aforementioned monoliths. Anodic LSV was performed on three‐electrode cells (Bio‐Logic VMP3) from the open‐circuit potential to 6 V versus Li^+^/Li (at 1 mV s^−1^). Cathodic LSV was similarly performed to −0.3 V versus Li^+^/Li. The working electrode was a stainless steel plate, the counter electrode was a piece of lithium, and the reference electrode was lithium. To fit the dimensions of the cell, 400 µL precursor solution was impregnated into a glass fiber separator (EL‐CELL, 18 mm diameter and 1.55 mm thickness) and then treated as mentioned above.

Electrode slurries were prepared by mixing 85 wt% NMC811 (MTI), 10 wt% carbon black (Super C65, Imerys), and 5 wt% poly(vinylidene fluoride) (PVDF, Alfa Aesar) in N‐methyl‐2‐pyrrolidone (NMP, Alfa Aesar, 99.0%+) using a planetary vacuum mixer (Thinky ARV 310 LED, 2000 rpm, 10 min). The slurries were coated on 15 µm thick aluminum foil (blade height 100 µm). Subsequently, the coated slurries were dried at 110 °C in a vacuum oven overnight. The active mass loading  was 2.8 mg cm^−2^, which corresponds to a theoretical capacity of 0.56 mAh cm^−2^ assuming a theoretical capacity of 200 mAh g^−1^.

For cell assembly, the electrode punches (15 mm diameter) were first impregnated with 50 µL precursor solution (corresponding to 10 µL mg^−1^ active material). After a few minutes, the wet electrodes were covered with a thin glass fiber separator (EL‐CELL, 19 mm diameter and 0.26 mm thickness) and 50 µL additional precursor solution was added to soak the glass fiber. The ensembles were then treated similarly as described above. After drying, NMC811/Li coin cells (type CR2025) were assembled in an argon‐filled glovebox (MBraun, H_2_O < 0.1 ppm, O_2_ < 0.1 ppm). After a 16 h resting period, the coin cells underwent galvanostatic cycling in a BCS‐805 (Bio‐Logic) battery tester in the voltage window of 3−4.4 V versus Li^+^/Li. Electrochemical impedance spectra were recorded over a frequency range of 10 kHz−10 mHz (10 mV amplitude).

## Conflict of Interest

A patent application related to this work has been filed as WO2023222511A1.

## Supporting information



Supporting Information

## Data Availability

The data that support the findings of this study are available from the corresponding author upon reasonable request.
