# Supplementary material for: Organic‐Inorganic Hybrid Solid Composite Electrolytes for High Energy Density Lithium Batteries: Combining Manufacturability, Conductivity, and Stability
Source: Adv Sci (Weinh). 2024 Nov 14;11(47):2406774. doi: 10.1002/advs.202406774 (PMC11653716; doi:10.1002/advs.202406774)
Supplement: Supplementary file 1 — Supporting Information [file ADVS-11-2406774-s001.pdf]

ADVANCED SCIENCE
Open Access

# Supporting Information

for *Adv. Sci.*, DOI 10.1002/advs.202406774

Organic-Inorganic Hybrid Solid Composite Electrolytes for High Energy Density Lithium Batteries: Combining Manufacturability, Conductivity, and Stability

*Dries De Sloovere*, Jonas Mercken, Jan D'Haen, Elien Derveaux, Peter Adriaensens, Philippe M. Vereecken, Marlies K. Van Bael and An Hardy**

# Supporting Information

## Organic-inorganic hybrid solid composite electrolytes for high energy density lithium batteries: combining manufacturability, conductivity, and stability

*Dries De Sloovere,[*] Jonas Mercken, Jan D'Haen, Elien Derveaux, Peter Adriaensens, Philippe M. Vereecken, Marlies K. Van Bael, An Hardy[*]*

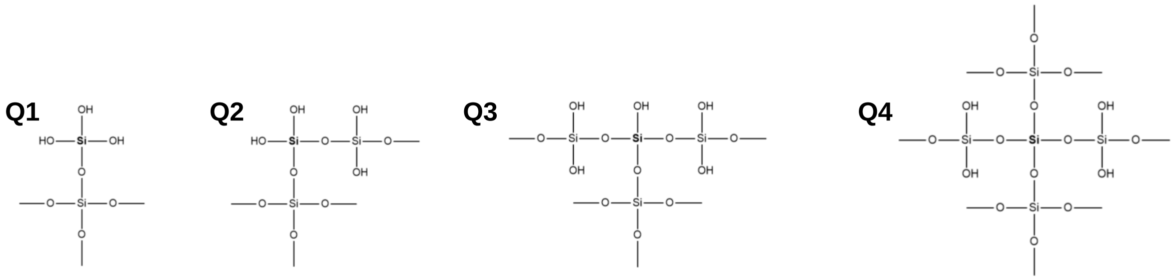


**Figure S1.** T structures and Q structures.

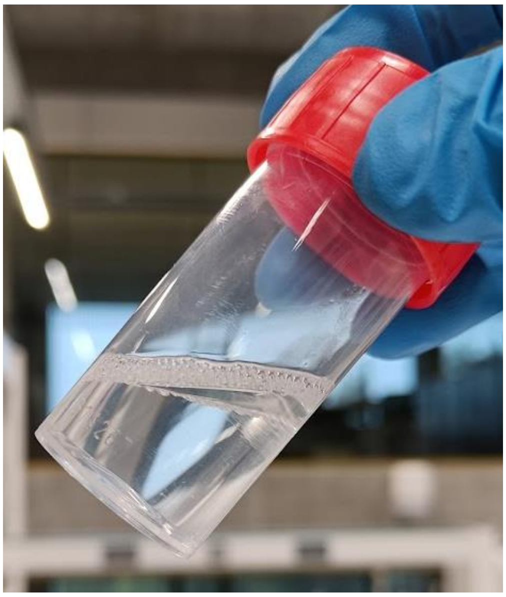

**Figure S2.** Photograph of precursor solution B after ~1 year of aging.

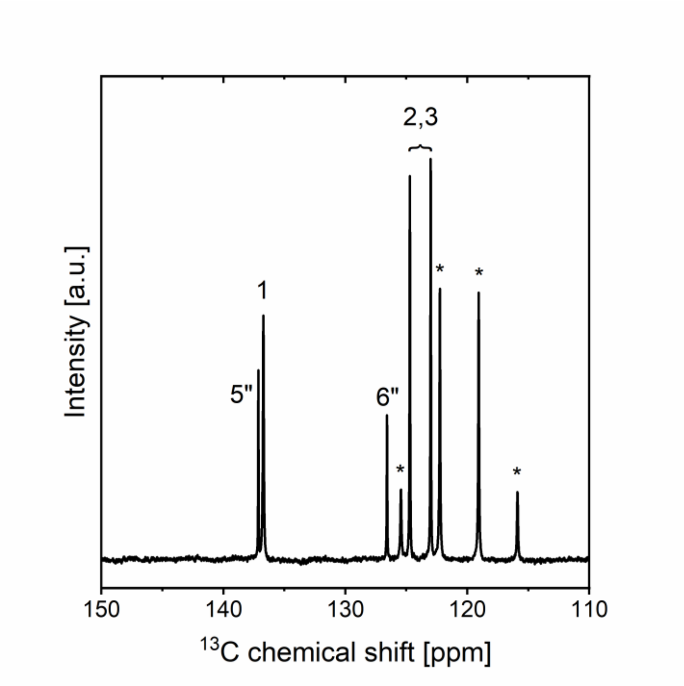


**Figure S3.** Excerpt of the $^{13}C$ NMR spectrum of precursor solution B directly after mixing.

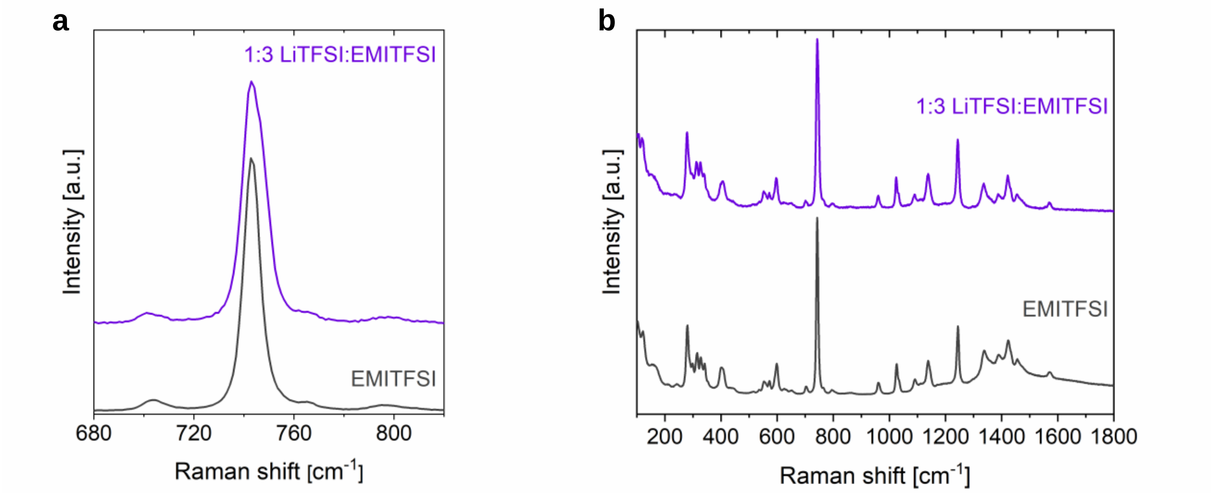


**Figure S4.** The Raman spectra of EMITFSI and 1:3 LiTFSI:EMITFSI. (a) excerpt, (b) full spectrum.

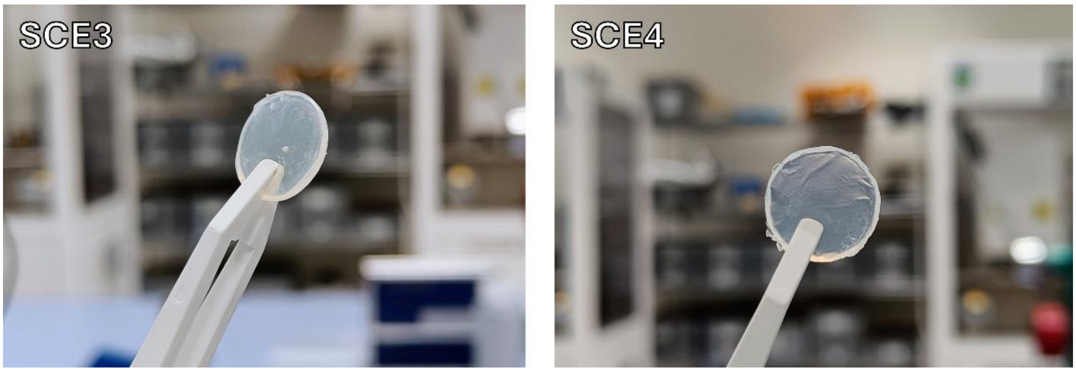


**Figure S5.** Photographs of SCE3 and SCE4.

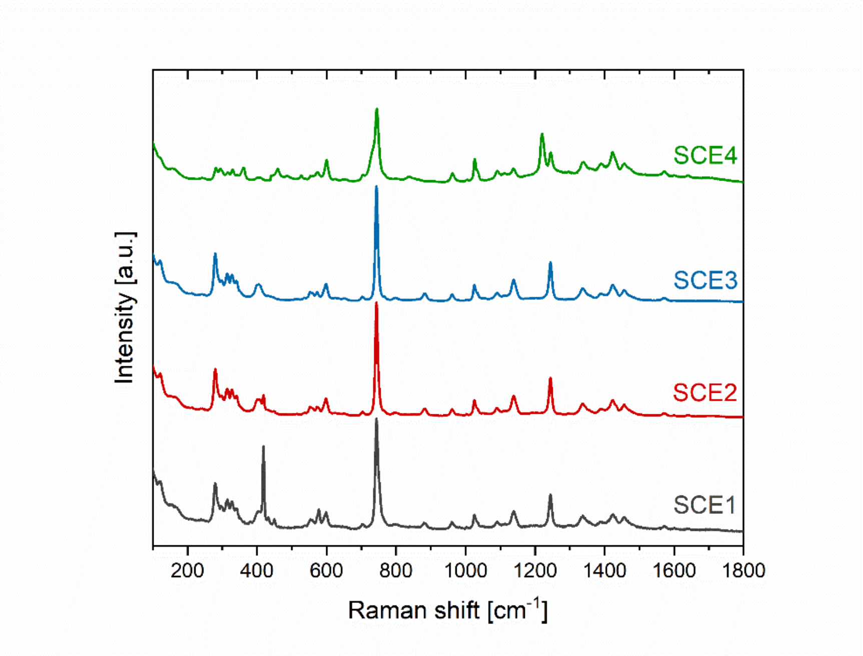


**Figure S6.** The complete Raman spectra of (dried) SCE1, SCE2, SCE3, and SCE4.

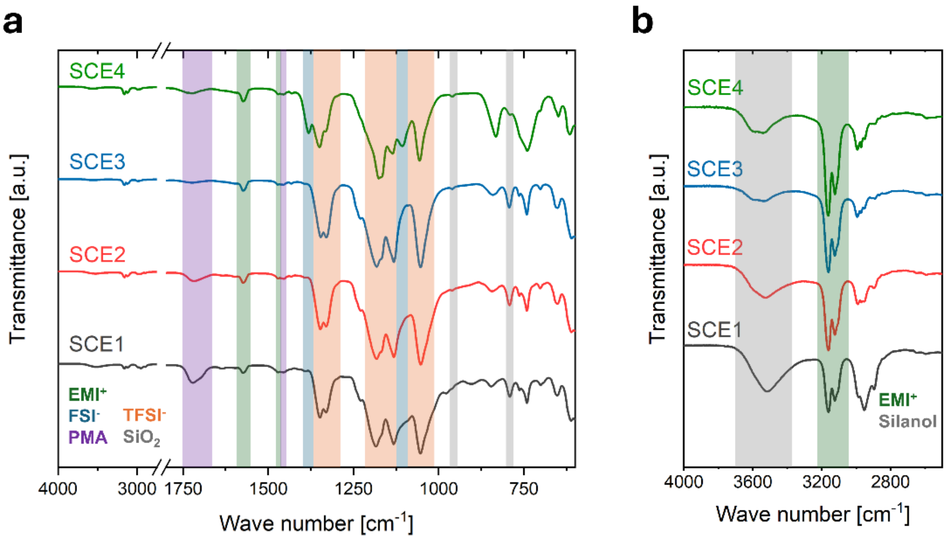


**Figure S7.** (a) The complete ATR-FTIR spectra of (dried) SCE1, SCE2, SCE3, and SCE4. (b) Excerpt of the same spectra. PMA refers to the propyl methacrylate fraction of TMSPMA.

The FTIR spectra of the dried SCEs are dominated by the signals relating to the incorporated ILs (i.e., of the $EMI^+$, $TFSI^-$, and $FSI^-$ anions) (Figure S7a). A number of less intense signals relates to the matrix. Particularly the stretching vibration of the TMSPMA carbonyl group is clear (~1720 $cm^{-1}$), along with some smaller signals relating to $SiO_2$. The presence of silanol groups is apparent from the broad bands at ~3600 $cm^{-1}$ (Figure S7b). For SCE1, there is one band, whereas two bands can be observed for the other SCEs. This may indicate a strong interaction of the silanol groups with the ILE in the latter SCEs.

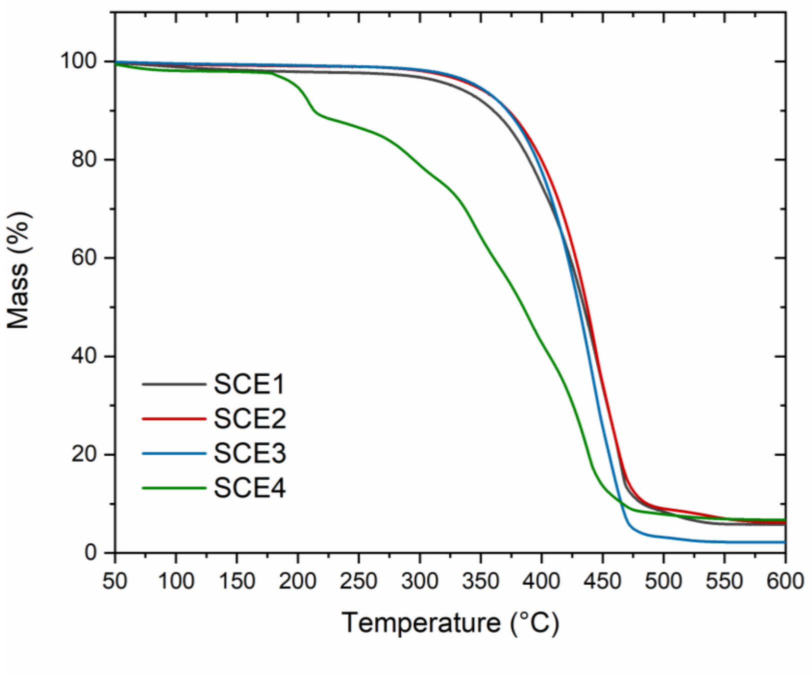


**Figure S8.** Thermogravimetric profiles of SCE1, SCE2, SCE3 and SCE4 recorded under dry air atmosphere with a heating rate of 10 °C $min^{-1}$.

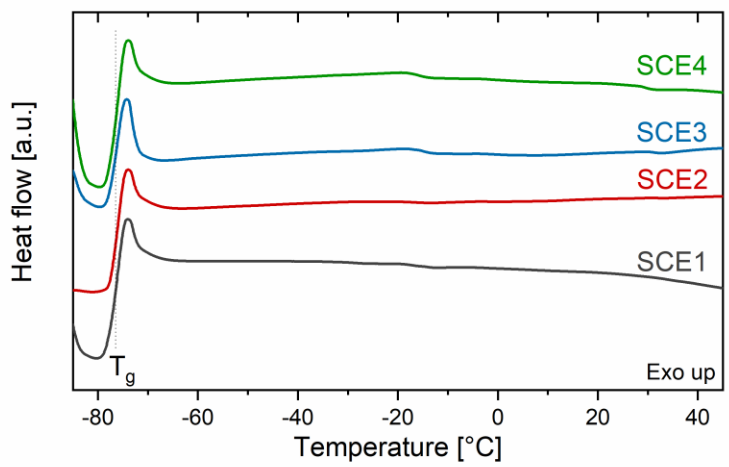


**Figure S9.** Heating cycles of SCE1, SCE2, SCE3 and SCE4 measured with DSC.

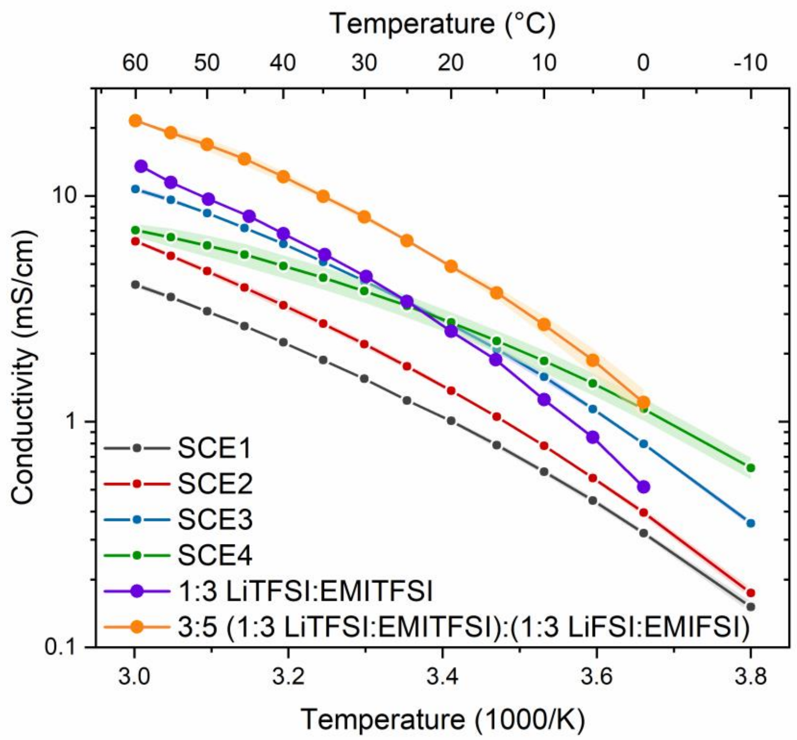


**Figure S10.** Arrhenius plots of the ionic conductivity of the SCEs and of the incorporated ILEs. Data points, which are the average of three separate measurements, are represented by dots, whereas the lines are only meant to guide the eye.

The VTF equation:

$$\sigma = \sigma_0 \exp\left(\frac{-B}{T-T_0}\right) \qquad \text{(Eqn. 1)}$$

Here, the pre-exponential factor represents the conductivity at infinite temperature. The pseudoactivation energy B along with the Vogel temperature $T_0$, characterizes the temperature dependence. The parameter fits for the SCEs are given in Table S1. The fitted parameter values allow for the interpolation of the conductivity within the studied temperature range. However, they do not accurately describe the properties of the SCEs outside this range.

**Table S1**. Parameters of the VTF equation fit to the conductivity data as a function of temperature.

| | $\sigma_0$ [mS cm$^{-1}$] | B [K] | $T_0$ [K] |
|---|---|---|---|
| SCE1 | 243 | 279 | 176 |
| SCE2 | 539 | 303 | 177 |
| SCE3 | 428 | 230 | 189 |
| SCE4 | 73 | 137 | 197 |
| 1:3 LiTFSI:EMITFSI | 491 | 197 | 207 |
| 3:5 (1:3 LiTFSI:EMITFSI):(1:3 LiFSI:EMIFSI) | 566 | 180 | 206 |

**Lithium plating/stripping**

As a reference, a symmetric Li/Li cell containing the ILE (1:3 LiTFSI:EMITFSI soaked in a glass fiber separator) was galvanostatically cycled at 0.05 mA cm$^{-2}$ (Figure S11). During the beginning of cycling, the overpotential is slightly lower compared to the cell containing SCE3, in line with the better electrolyte/lithium contact. However, a sudden drop in overpotential can be observed after 45 h. This indicates a soft short, i.e. the growth of lithium metal through the glass fiber separator and the formation of a direct electronic pathway between the electrodes. Therefore, these results indicate that SCE3 suppresses dendrites better than the ILE.

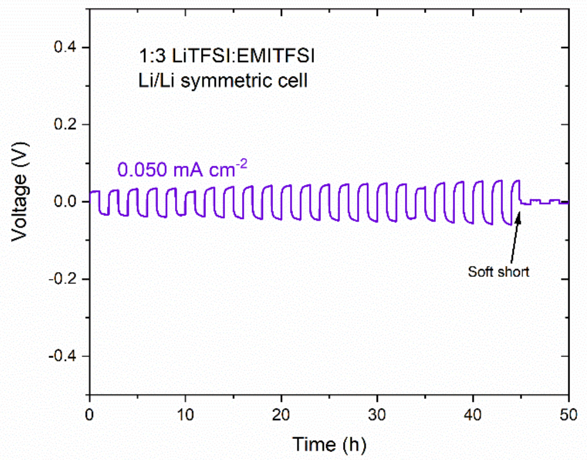


**Figure S11.** Galvanostatic cycling of a symmetric Li/ILE/Li cell at a current density of 0.05 mA cm$^{-2}$ (0.05 mAh cm$^{-2}$ per half cycle).

**SEM-EDX**

The impregnation of the SCE precursor solutions into porous electrodes was investigated by SEM-EDX on thick electrodes (130 µm, i.e., significantly thicker than the electrodes used for electrochemical investigation). More specifically, an EDX line scan was performed from the top of the electrode to the current collector (Figure S12). Due to the porosity of the electrode,

the signal intensity for oxygen and transition metals is high when NMC811 particles are scanned. More importantly, the sulfur signal remains low throughout the entire electrode. When the electrode is impregnated with SCE3 (by drop casting 50 µl precursor solution and irradiating with UV light), the sulfur signal becomes dominant over the whole electrode, except when an NMC811 particle is being scanned (Figure S13). This indicates that the SCE is indeed present throughout the whole electrode. The same conclusion can be reached for the electrode impregnated with SCE4 (Figure S14). In this case, however, the electrode retains some porosity, presumably because of the higher volumetric content of volatiles in the precursor solution. In any case, these results indicate that the SCE precursor solutions can be readily impregnated into porous electrodes with thicknesses at least up to 130 µm.

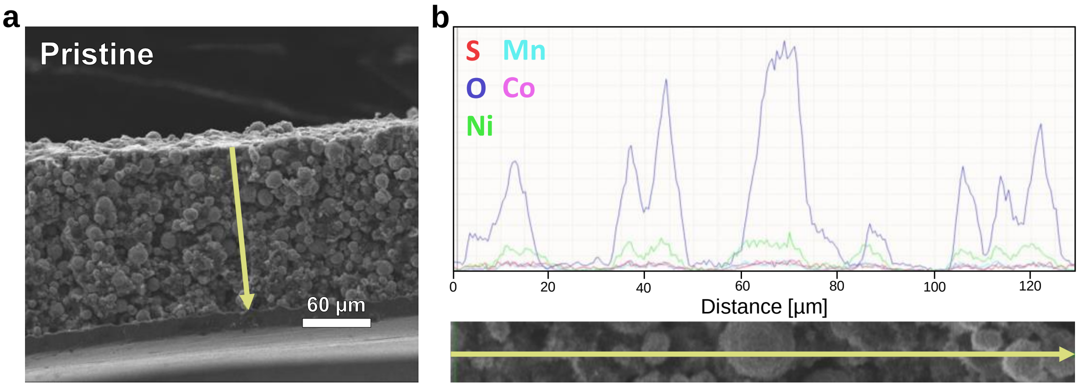


**Figure S12.** (a) SEM image of an unimpregnated thick NMC811 electrode. The arrow indicates the line along which the line scan was taken. (b) EDX line scan along with an excerpt of the SEM image.

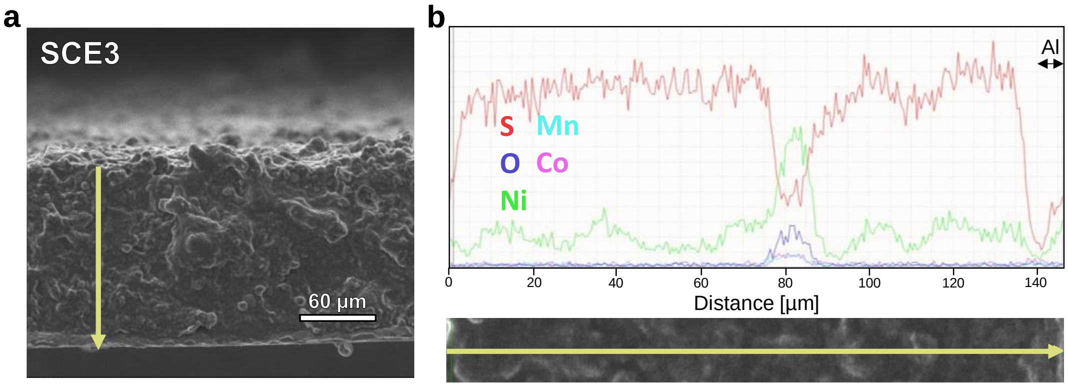


**Figure S13.** (a) SEM image of a thick NMC811 electrode impregnated with SCE3. The arrow indicates the line along which the line scan was taken. (b) EDX line scan along with an excerpt of the SEM image. During the impregnation procedure, 50 µl of the precursor solution was dropcasted on an electrode punch with 15 mm diameter. This was immediately followed by 1 hour of UV irradiation. Finally, the ensembles were dried in a dry room.

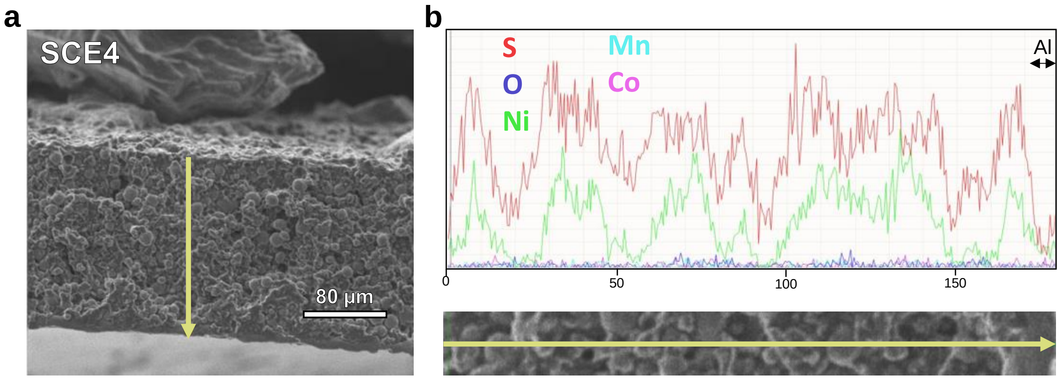


**Figure S14.** (a) SEM image of a thick NMC811 electrode impregnated with SCE4. The arrow indicates the line along which the line scan was taken. (b) EDX line scan along with an excerpt of the SEM image. During the impregnation procedure, 50 µl of the precursor solution was dropcasted on an electrode punch with 15 mm diameter. This was immediately followed by 1 hour of UV irradiation. Finally, the ensembles were dried in a dry room.

NMC811/SCE3/Li and NMC811/SCE4/Li cells were constructed and subjected to 25 cycles at 0.1 C (3V – 4.4 V). Afterwards, the cells were disassembled and the lithium foil was studied using SEM-EDX. Whereas pristine lithium is relatively smooth (Figure S15a), the cycled lithium foils are covered with a tightly adhered layer (Figure S15b, Figure S15c). The presence of glass fibers (originating from the glass fiber separator) throughout the surface indicates that the electrolyte is tightly attached to the surface of the lithium foil. Darker patches are visible on both cycled lithium foils, and EDX indicates that these are holes in the adhered layers (Figure S15d). This implies that the surface morphology of the cycled lithium metal foils cannot be accurately investigated.

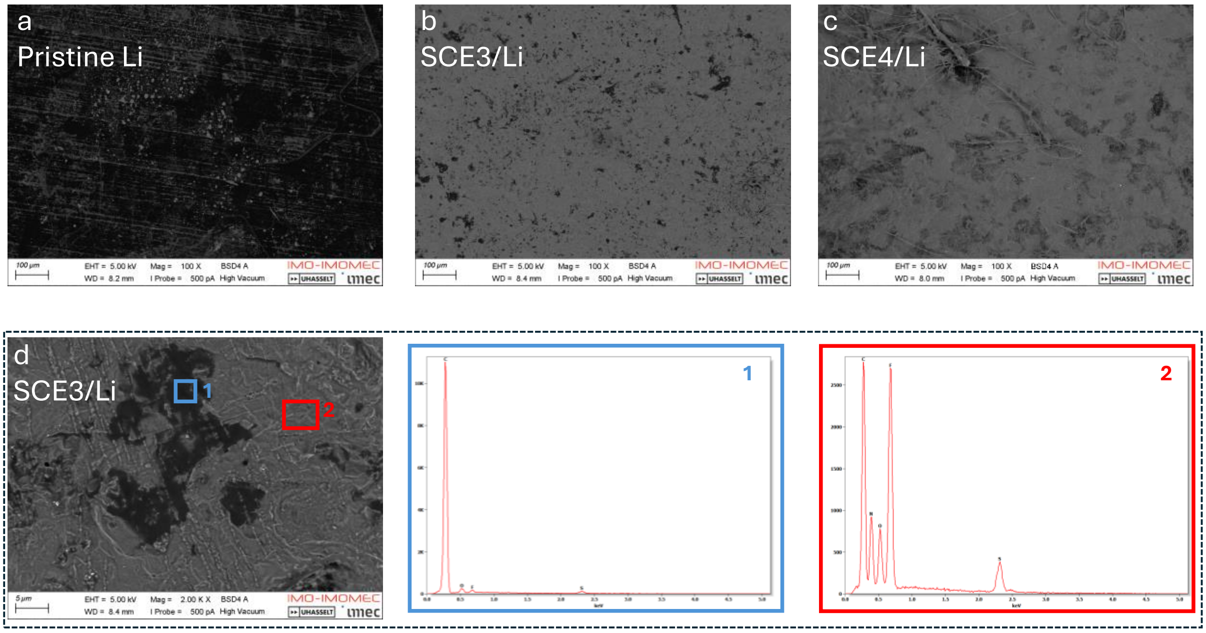


**Figure S15.** SEM images of (a) lithium foil, (b) lithium foil extracted from a NMC811/SCE3/Li coin cell subjected to 25 cycles (0.1 C, 3 - 4.4 V vs $Li^+$/Li), (c) lithium foil extracted from a NMC811/SCE4/Li coin cell subjected to 25 cycles (0.1 C, 3 - 4.4 V vs $Li^+$/Li), (d) lithium foil extracted from a NMC811/SCE3/Li coin cell subjected to 25 cycles (0.1 C, 3 - 4.4 V vs $Li^+$/Li), where spots 1 and 2 indicate the locations where EDX spectra were taken.
